# Diagnosis of *mycoplasma pneumoniae* by loop-mediated isothermal amplification: systematic review and meta-analysis

**DOI:** 10.1186/s12879-019-3799-4

**Published:** 2019-02-19

**Authors:** Zi-Hong Cai, Yuan-Yuan Dai, Lin-Yan Huang, Wen-Sheng Zhang, Xu-Guang Guo

**Affiliations:** Department of Clinical Laboratory Medicine, the Third Affiliated Hospital of Guangzhou Medical University, Guangzhou, Guangdong China

**Keywords:** *Mycoplasma pneumoniae*, Mycoplasma pneumonia, Loop-mediated isothermal amplification, PCR

## Abstract

**Background:**

A novel method, termed loop-mediated isothermal amplification (LAMP), was developed by Notomi et al. (2000). Individually published results have been reported that this technology has been successfully applied to the detection of a variety of pathogens. However, the overall diagnostic accuracy of LAMP for *Mycoplasma pneumoniae* (MP) remains unclear. A meta-analysis was therefore performed to review the accuracy of LAMP for *Mycoplasma pneumoniae.*

**Methods:**

Cochrane Library and PubMed were systematically searched and checked for studies using LAMP for detecting *mycoplasma pneumoniae.* We used PCR as a reference standard to evaluate the quality of the studies eligible for inclusion in the meta-analysis. Then, the data from the studies were extracted by two independent assessors. Meta-DiSc 1.4 software was utilized to test the heterogeneity of sensitivity (SEN), specificity (SP), positive likelihood ratio (PLR), negative likelihood ratio (NLR), and diagnosis odds ratio (DOR). The pooled analysis results were plotted, and the summary receiver operating characteristic (SROC) curve was plotted by calculating the area under the curve (AUC). Generated pooled summary estimates (95% CIs) were calculated for the overall accuracy, and a bivariate meta-regression model was used for the meta-analysis.

**Results:**

Seven studies with nine fourfold tables were included in this meta-analysis. The pooled SEN and SPE for diagnosing *Mycoplasma pneumoniae* were 0.90 (95% CI: 0.87–0.93) and 0.98 (95% CI: 0.96–0.99), respectively. The PLR was 31.25 (95% CI: 14.83–65.87), NLR 0.10 (95% CI: 0.05–0.22), DOR 399.32 (95% CI: 172.01–927.00), and AUC 0.9892.

**Conclusions:**

In conclusion, compared with PCR, LAMP is a valuable alternative method for *Mycoplasma pneumoniae* diagnosis in clinic with high sensitivity and specificity. However, more evidence is required to confirm that LAMP can fully replace other methods in the clinical diagnosis of MP.

## Background

*Mycoplasma pneumoniae* (MP) is the major pathogen causing human *Mycoplasma pneumonia*, which accounts for approximately 20–30% of all types of *pneumonia* worldwide [1, 2]. Prospective investigations support the notion that it has been the most common bacterial etiology in upper respiratory tract infections [3]. MP infection not only causes upper and lower respiratory disease, but also affects other systems of the human body, whose severity ranges from mild to life-threatening [4–9].The laboratory identification of the etiology of such organisms that lack a cell wall is critical to the determination of the correct course of treatment. Three common methods exist for the identification of MP infection: culture separation, serological tests, and PCR. Obviously, the gold standard for diagnosis of MP infection is MP culture separation. However, culture is not routinely applied for MP diagnosis in clinic due to the need for special medium, extensive time consumption, cumbersome experimental conditions, a relatively lower detection rate, and the delayed confirmation of MP infection, which is generally too slow to be of practical use. Another reliable detection method, PCR [10], is more widely implemented for diagnosis because it is more time-efficient and sensitive. Nevertheless, it also requires expensive equipment, molecular biology facilities, and experienced experimenters, which are not always available in smaller laboratory. As a consequence, PCR is not appropriate for application in primary hospitals, too. Serological tests diagnosis is frequently invalid because the tests are usually positive one–two weeks or more after the disease onset [11], and thus it is difficult to implement the results of such testing to actual treatment decisions.

In 2000,Notomi et al. developed a method with high specificity and efficiency to amplify DNA rapidly under isothermal conditions, which was called loop-mediated isothermal amplification [12].Many reports confirmed the successful application of this technology to the detection of a variety of pathogens [13–16].Additionally, according to previous reports, the LAMP assay has sensitivity for detecting MP that is almost identical to that of PCR [17, 18] and is simpler to operate and less expensive than PCR. However, the overall diagnostic accuracy of LAMP for *Mycoplasma pneumoniae* remains unclear. The main purpose of the present study was to validate that LAMP is a more congruent method for application of the results obtained to actual treatment decisions in both superior and primary hospitals.

## Methods

### Electronic searches

We conducted a full search on PubMed and the Cochrane library from January 2000 to September 2018 using the keywords *Mycoplasma pneumoniae*, LAMP. The following search stratagem was utilized: ((*Mycoplasma pneumoniae*) AND (loop-mediated isothermal amplification OR LAMP)). The search was supplemented by inspection of the bibliographies of the articles retrieved. No language restrictions were applied to the search

### Study screening and selection

Two investigators independently screened the potentially relevant publications by examining the full-text. Upon completion of the independent screening, two investigators cross-checked the results and discussed the existing differences, followed by making a decision on whether to include them or not. If the results were inconsistent, third investigators were included and all assessed the results together. All studies meeting the eligibility criteria were included in this analysis

### Inclusion and exclusion criteria

Studies were included if (i) human samples were analyzed; (ii) diagnostic accuracy studies that compared LAMP with PCR for detecting MP; the latter were employed as the gold standard; (iii) the generated data were sufficient to construct 2•2 tables for calculating the sensitivity, specificity, and likelihood ratios

Studies were not included if: (i) The sample were from animals or other species; (ii)The gold standards were not used among the PCR samples— assayed for the detection of MP, or the study did not implement a LAMP assay; (iii) Possible duplicate publications, when an author published it more than once.

### Data extraction

The extracted data included the title of the article, author, publication year, country, the number of included specimens, gold standard, detection method, true positive, false positive, true negative, and false negative. We extracted the data and constructed the 2•2 table.

### Quality assessment

Two researchers independently assessed the articles included, using unified quality evaluation form, the Quality Assessment of Diagnostic Accuracy Studies (QUADAS-2) as a standard. The study QUADAS-2 quality criteria are given by Review Manager 5.2, which consists of four domains (patient selection, index test, reference standard, and flow and timing). These were used to assess the risk of bias, whereas the assessment of the first three was to be taken into consideration in the clinical applicability evaluation.

### Statistical analysis

Using meta-DiSc 1.4 software, two investigators independently analyzed the data recommended for meta-analysis of the diagnostic studies: Sensitivity, specificity, positive likelihood ratio (PLR), negative likelihood ratio (NLR), diagnostic odds ratio (DOR), and 95% confidence intervals (CI). If this information was not available, the respective values were calculated from the data provided in the article. We calculated and analyzed spearman correlation coefficient to estimate the heterogeneity of the studies, including the threshold and the nonthreshold effects. When a heterogeneity effect was present, the combined statistics were calculated using the random-effect model. When no heterogeneity effect was available, the fixed-effect model was implemented

## Results

### Characteristics of the included studies

**Fig. 1:**
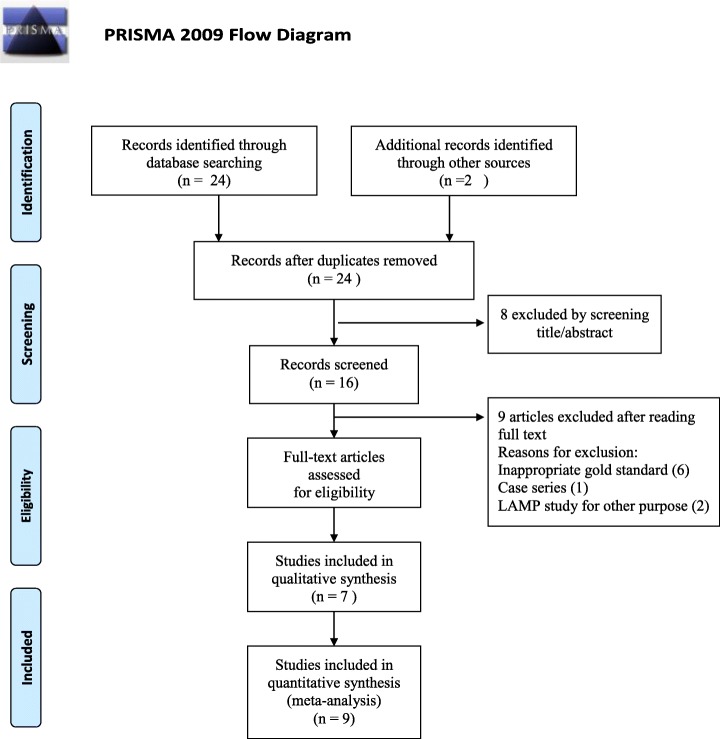
Flow diagram of study identification and inclusion. *From:* Moher D, Liberati A, Tetzlaff J, Altman DG, The PRISMA Group (2009). *P*referred *R*eporting *I*tems for *S*ystematic Reviews and *M*eta- *A*nalyses: The PRISMA Statement. PLoS Med 6(7): e1000097. 10.1371/journal.pmed1000097. For more information, visit www.prisma-statement.org

Based on the search stratagem specified earlier, we obtained a total number of 26 articles, which decreased to 24 after the duplicates were removed. Then, by screening the titles and abstracts, and excluding the ineligible studies, we finally included seven articles, (Fig. 1), which were selected for full-text review and meta-analysis [18–24]. From these seven articles, we extracted nine groups of data, consisting of a total number of 1325 samples. The characteristics of these studies are summarized in Table 1.

**Table 1:** Characteristics of the included studies

Studies included	Country	Mycoplasma pneumonia patients	Control group	Mycoplasma pneumonia patients included in the staging	Detection method	Company of the reagent source	Gold standard	Blind	Value of the positive judgment	Results			FN
										TP	FP	TN	
Ryoichi Saito, 2005	Japan	6	89	No	LAMP	1.Roche Diagnostics	Real-time PCR	yes	2 × 10 ^2^	6	0	89	0
						2.Qiagen							
						3.Applied Biosystems							
FEI ZHAO, 2013	China	21	59	No	LAMP	Qiagen	Real-time PCR	yes	unclear	21	5	54	0
Amy E. Ratliff II, 2014	American	22	192	No	LAMP	1.Qiagen	Real-time PCR	yes	*C*_*T*_ ≤ 42.0	22	2	190	0
						2.Agilent							
Brianna L. Petrone, 2015	American	226	26	No	LAMP	1.Roche Applied Science	Real-time PCR	yes	*CT < 30*	200	1	25	26
						2.Thermo Scientific							
						3.MP Biomedicals							
Brianna L. Petrone, 2015	American	39	33	No	LAMP	1.Roche Applied Science	Real-time PCR	yes	CT ≥ 30	32	0	33	7
						2.Thermo Scientific							
						3.MP Biomedicals							
Fujio Kakuya, 2017	Japan	58	43	NO	LAMP	1.Eiken Chemical	Real-time PCR	Yes	an MP DNA load of> = 4.5 copies/μL	15	0	43	0
						2.Asahi Kasei Pharma							
Fujio Kakuya, 2017	Japan	58	50	NO	LAMP	1.Eiken Chemical	Real-time PCR	Yes	an MP DNA load of> = 4.5 copies/μL	8	0	43	7
						2.Asahi Kasei Pharma							
Ho Namkoong, 2018	Japan	73	84	No	LAMP	1. Mizuho Medy	Real-time PCR	yes	unclear	70	1	83	3
						1. Fujifilm							
						2. Asahi Kasei Pharma							
Xin Yuan, 2018	China	116	9	No	LAMP	1. Oxoid	Real-time PCR	yes	unclear	37	6	3	79
						2. New England Biolabs							
						3. Da An Gene Co,							
						4. Sinopharm Chemical Reagents							
						5. Shanghai Simco Biotech							
						6. Beijing Mylab Corporation							

*TP* true positive, *FP* false positive, *FN* false negative, *TN* true negative

### Methodological quality

**Fig. 2:**
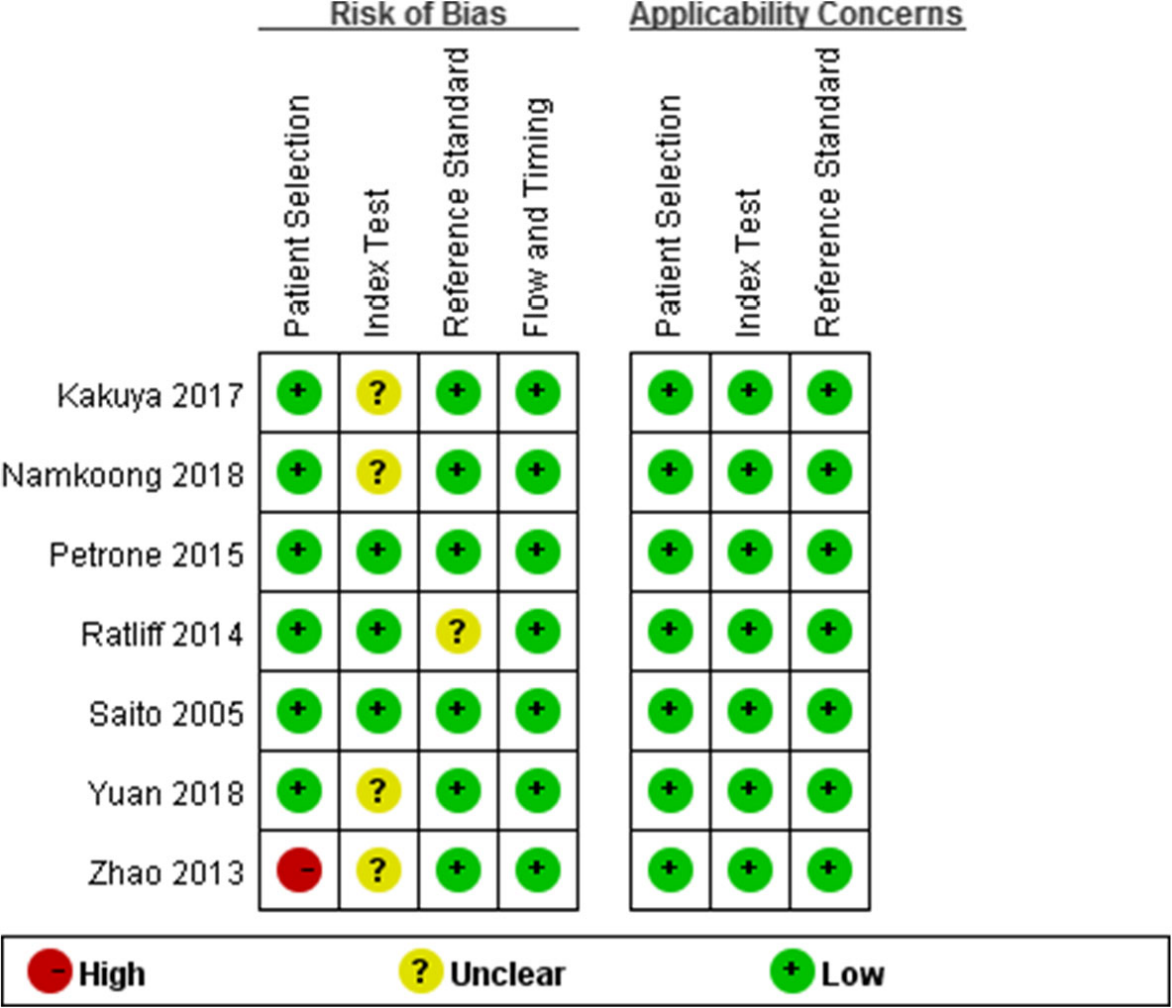
Quality evaluation of the included studies

**Fig. 3:**
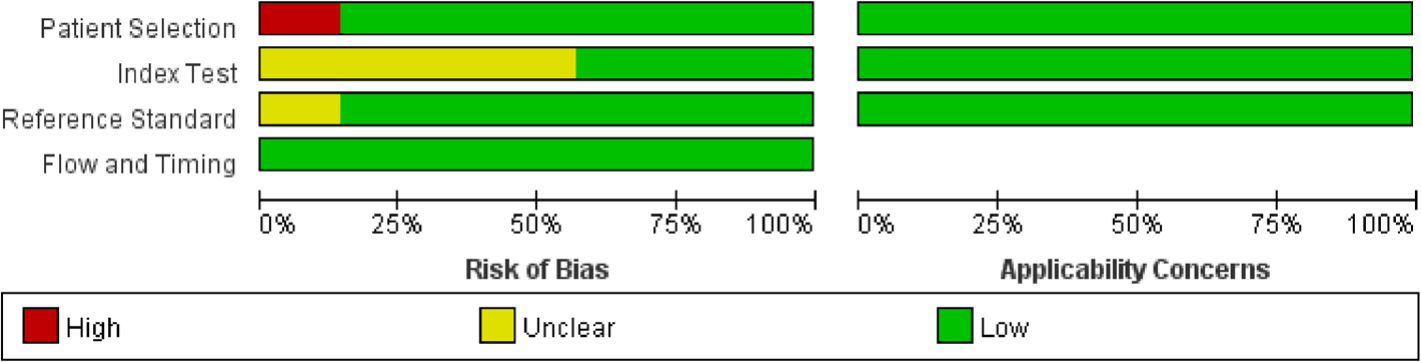
Risk of bias and applicability concerns graph: review authors’ judgments about each domain presented as percentages across the included studies

Using Review Manager 5.3, we drew a conclusion that most studies were at a low risk of bias and low concern regarding the applicability of their results. Nine studies meet all four domains of the criteria, whose quality assessment for the individual studies result are presented in Fig. 2. In addition, the overall risk of bias and applicability concerns for the seven selected articles can be seen in Fig. 3. In the patient selection domain, approximately 80% of the studies were at low risk of bias, because they enrolled participants consecutively and avoided inappropriate exclusions. The remaining study was graded as being at high risk of bias as it had a case-control design. In the index test domain, we considered the majority of studies to be at unclear risk of bias (60%), because most of the articles’ thresholds were unclear, or the index test was interpreted with knowledge of the results of the reference standard. The remaining studies were judged to be at low risk. In the reference standard domain, we assigned 80% of studies to be at low risk of bias, because it was stated that the reference standard results were interpreted without knowledge of the results of the index test. Applicability was of low concern for all studies in the reference standard domain. In the flow and timing domain, all studies were judged to be at low risk of bias because all patients were included in the analysis, the appropriate reference standard was used, and an appropriate interval between the index test and the reference standard was available.

### Publication Bias

**Fig. 4:**
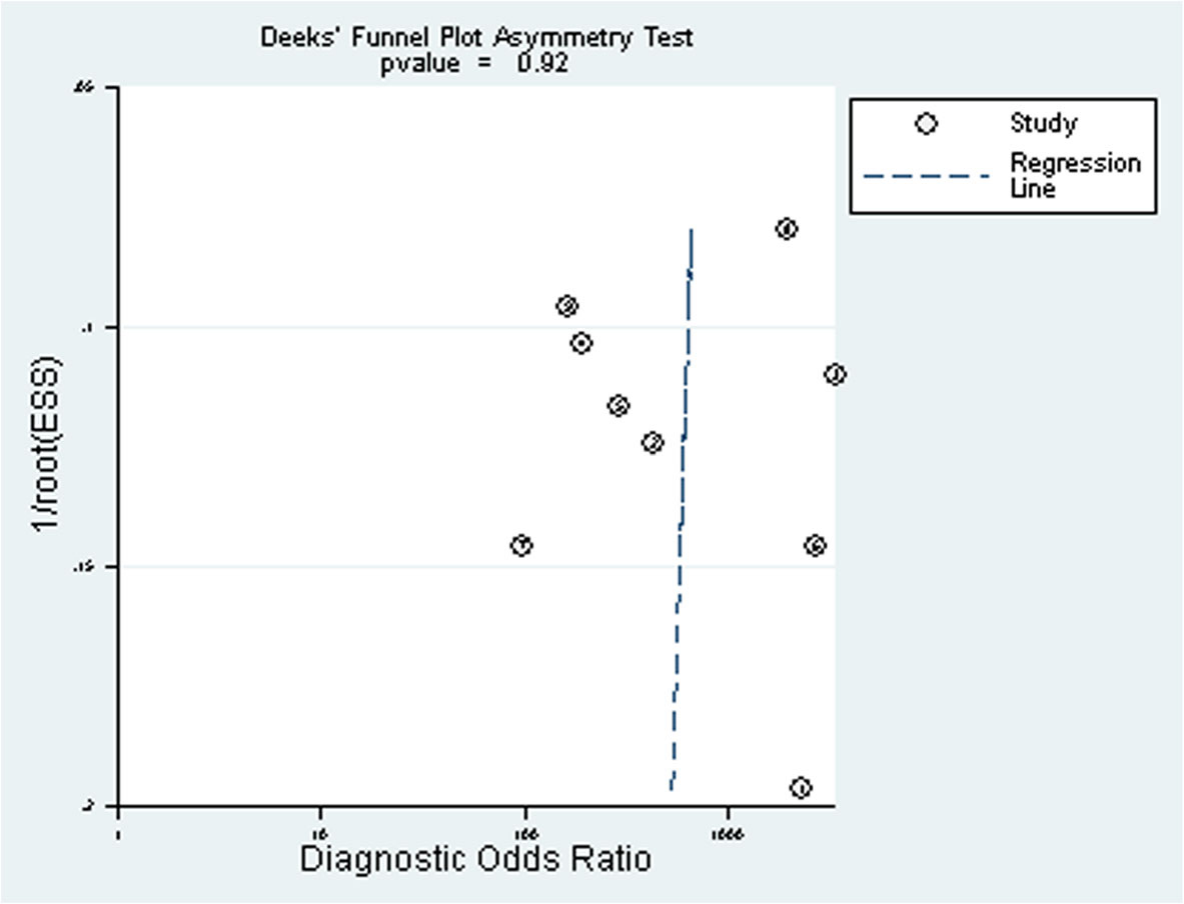
Deeks’ funnel plot asymmetry test to assess publication bias in estimates of diagnostic odds ratio for LAMP detection of mycoplasma pneumoniae infections

No publication bias was found in the Deek’s funnel plot (Fig. 4). The Egger test indicated that the current publication bias of the study was low (*P* = 0.261).

### Threshold effect analysis

**Fig. 5:**
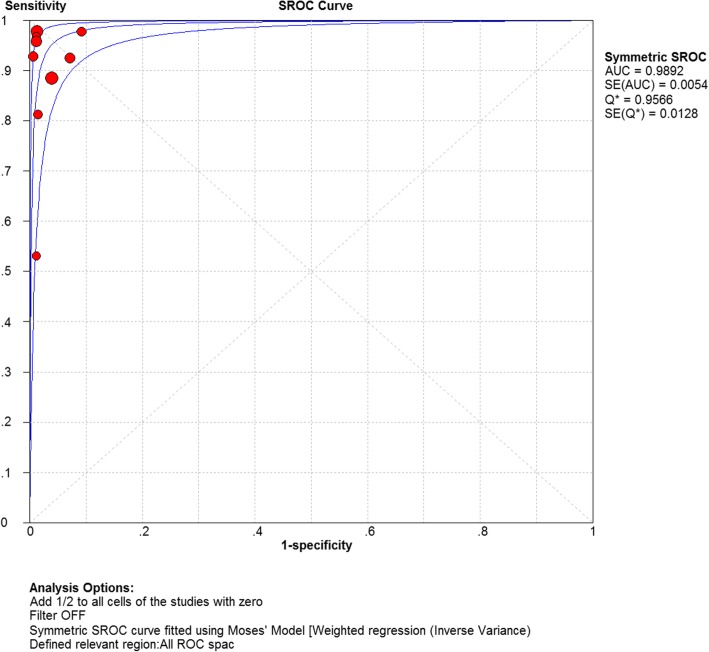
Summary receiver operating characteristic curves of mycoplasma pneumoniae infections detected by LAMP

In the analysis of the threshold effect, the Spearman correlation coefficient = 0.100; *P*-value = 0.797 (*P* > 0. 05) were applied. Moreover, we analyzed the SROC curve (Fig. 5), which was not characterized by a “shoulder arm” distribution. Thus, we concluded that no threshold effect was present among the articles included.

### Heterogeneity analysis of non - threshold effect

**Fig. 6:**
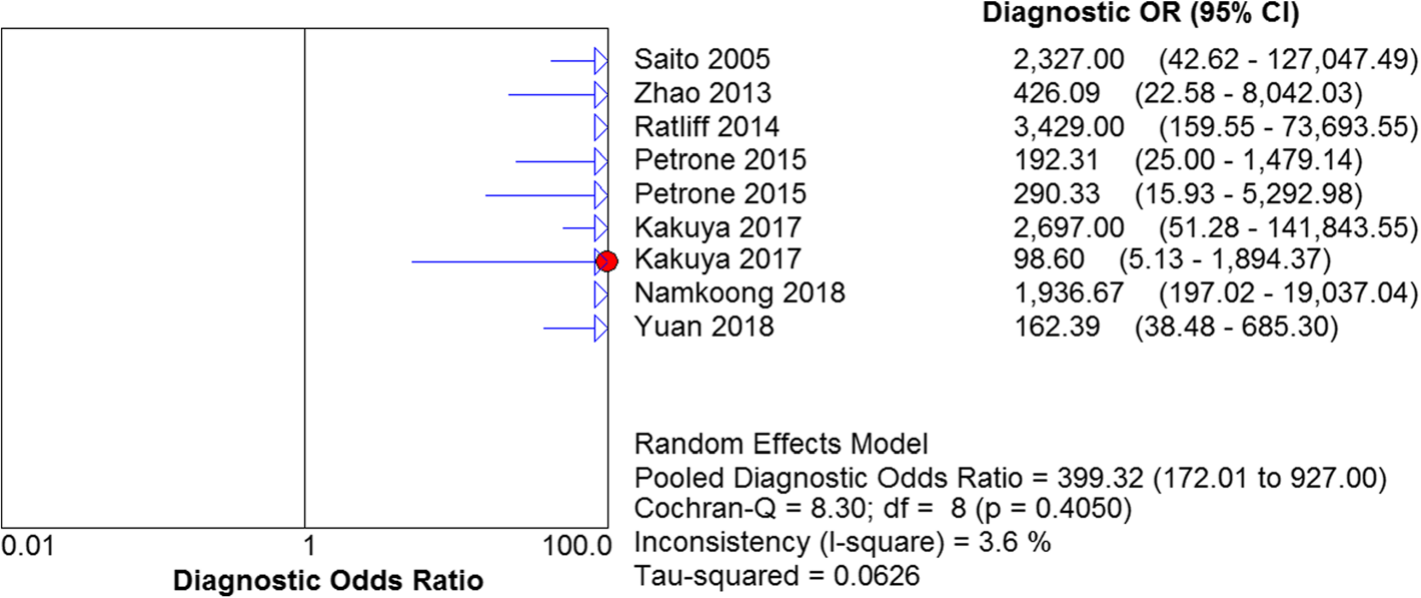
Forest plots for the combined diagnostic OR of LAMP

A forest map with a random pattern was used to draw the ratio. As can be observed in Fig. 6, the following values were obtained: Cochran-Q = 8.30, *P* = 0.4050 (*P* > 0. 05), and the inconsistency = 3.6% (inconsistency < 50%), indicating that the absence of heterogeneity in the non-threshold effect.

### SROC curve

A fixed-effect model was adopted to fit the SROC curve. As can be seen in Fig. 5, AUC = 0.9892, and the Q index was 0.9566 (SE = 0.0128). We, therefore, suggested that the detection of MP infection by LAMP was of high accuracy.

### Merge analysis results

**Fig. 7:**
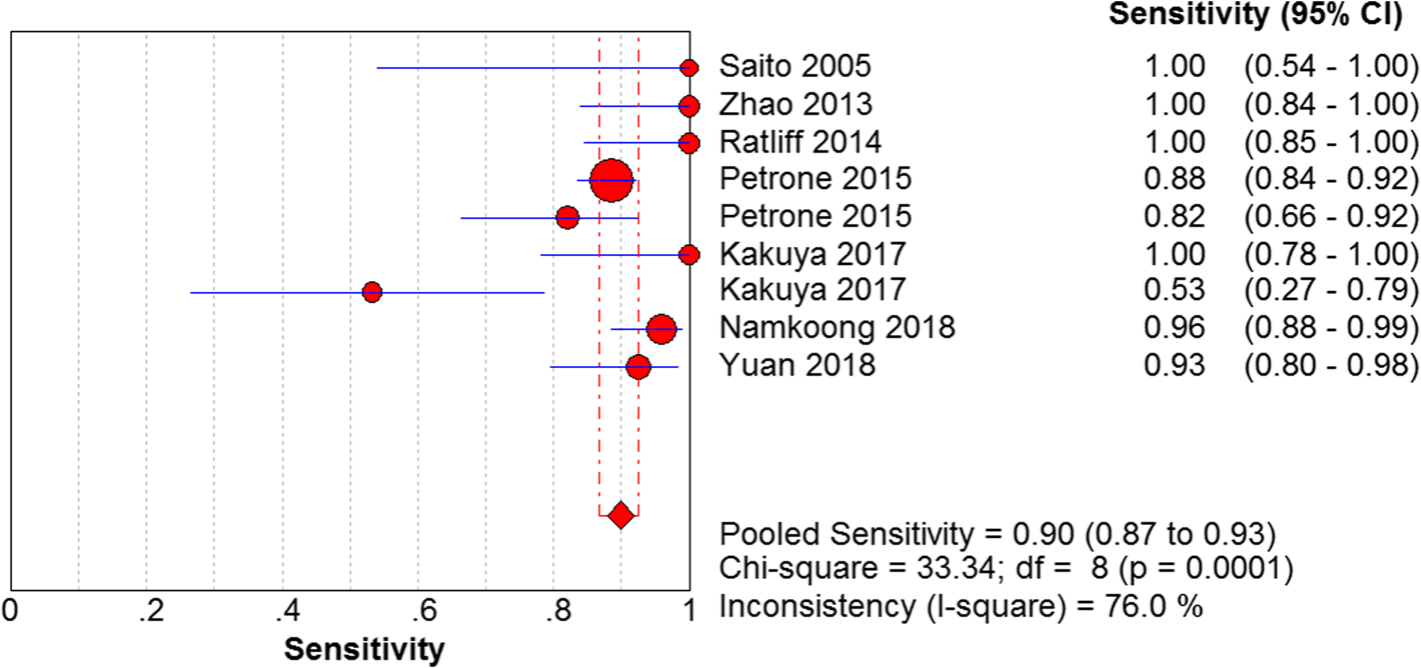
Forest plots for the combined sensitivity of LAMP

**Fig. 8:**
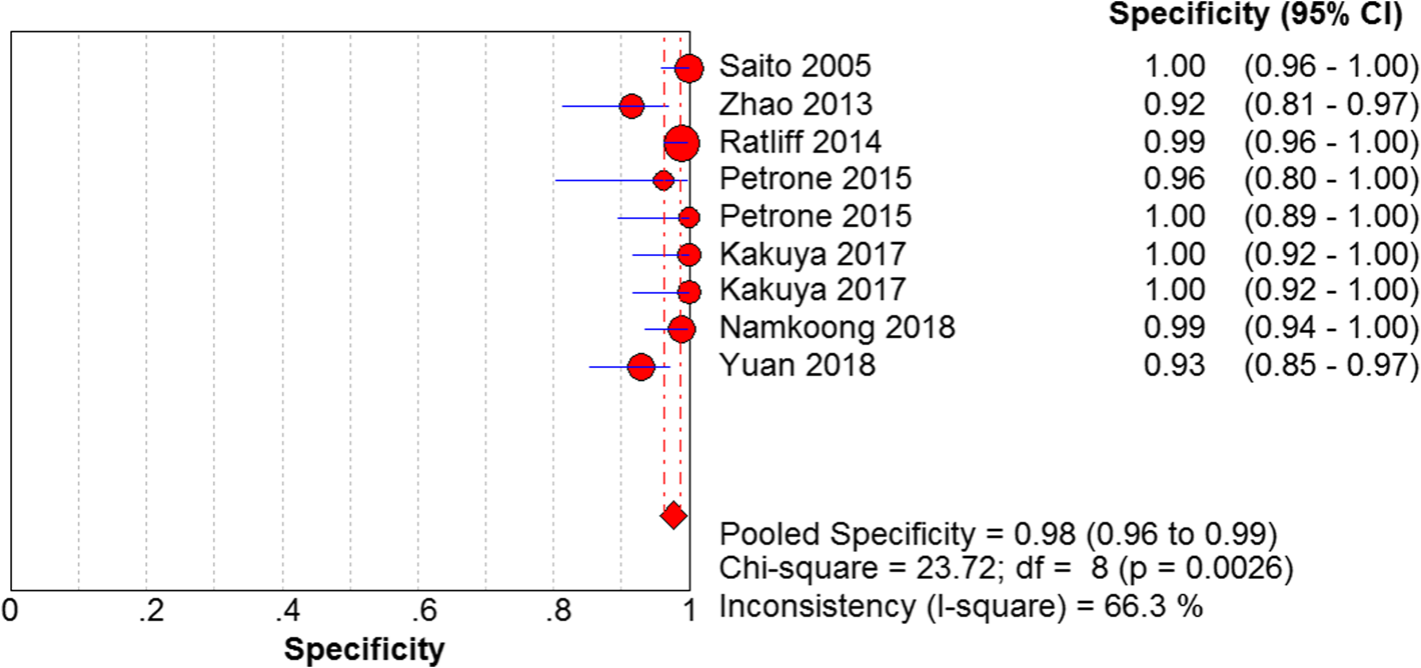
Forest plots for the combined specificity of LAMP

**Fig. 9:**
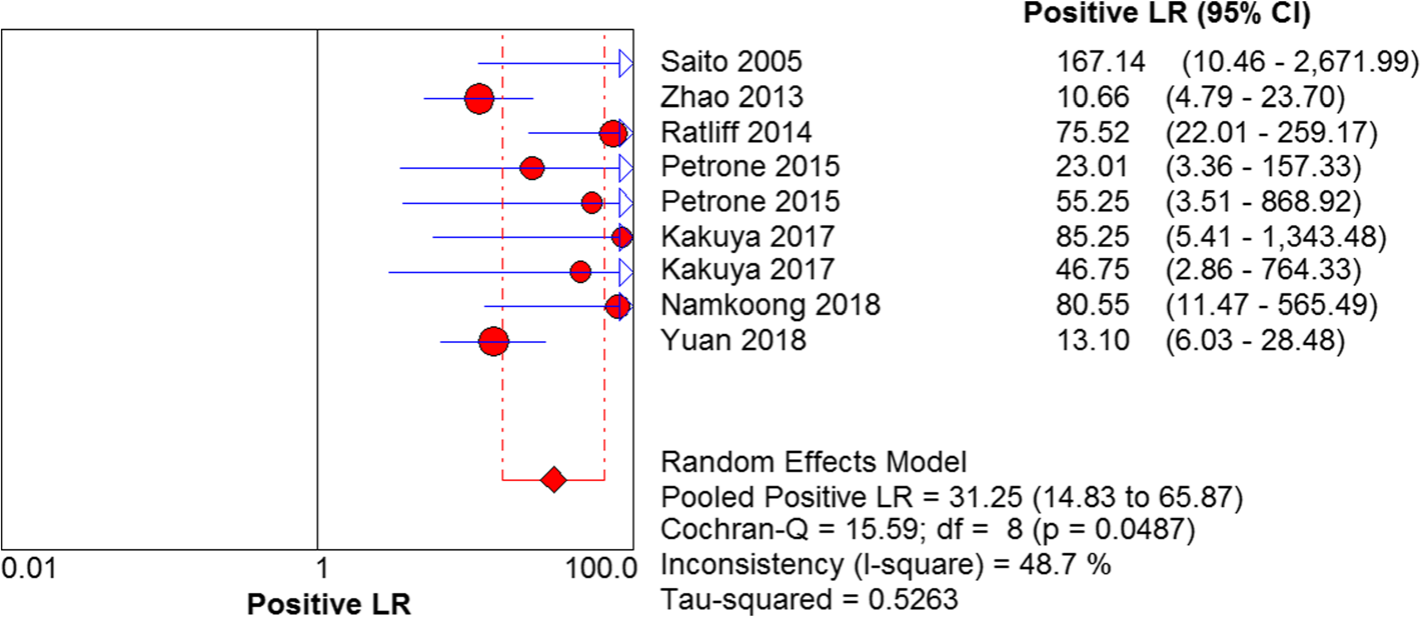
Forest plots for the combined positive LR of LAMP

**Fig. 10:**
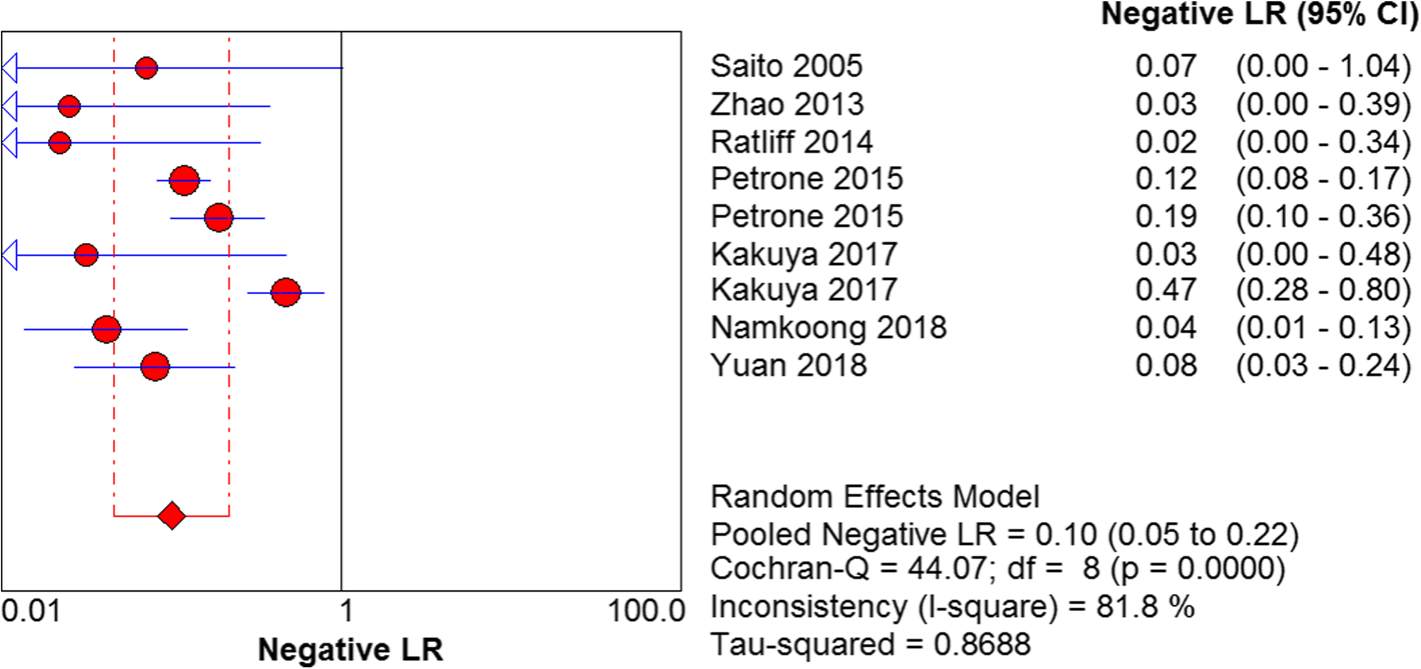
Forest plots for the combined negative LR of LAMP

The results are shown in Figs. 7, 8, 9 and 10. LAMP technology was employed to detect *mycoplasma pneumoniae* merger sensitivity, specificity, positive LR, and negative LR, whose values were respectively 0.90 (95% CI (0.87, 0.93), 0.98 (95% CI (0.96, 0.99)), 31.25(95% CI (14.83, 65.57)), and 0.10 (95% CI (0.05, 0.22)), correspondingly; the value of the pooled diagnostic odds ratio was 399.32 (95% CI (172.01, 927.00)].

## Discussion

In this study, we conducted a thorough search by the application of strict screening criteria and finally included seven articles. Nine 2*2 tables on the diagnosis of *Mycoplasma Pneumoniae* by LAMP were carried out. The results of the quality evaluation showed that the combined sensitivity and specificity of LAMP in the diagnosis of *Mycoplasma Pneumoniae* were 0.90 and 0.98; the negative likelihood ratio, positive likelihood ratio and diagnostic ratio were 0.10, 31.25, and 399.32, respectively. The SROC AUC was 0.9892, which indicated high sensitivity and specificity of LAMP in the diagnosis of *Mycoplasma Pneumoniae*. The SROC curve was close to the upper left corner; that is, the area under the curve was extremely large. Thus, LAMP is highly accurate in the diagnosis of *Mycoplasma Pneumoniae*. According to the analysis of the four studies included in the analysis conducted by the STATICS software, the bias coefficient was *P* = 0.108 > 0.05, indicating that the probability of publication bias was subtle.

PCR and culture are widely used as the gold standard for the detection of *Mycoplasma Pneumoniae*, but there are some limitations to their wide clinic application. First, the MP culture separation requires a special culture medium and a long time. Furthermore, the experimental protocol is cumbersome, resulting in a low detection rate. Hence the isolation and culture are not suitable for the early clinical diagnosis of MP infection. In addition, PCR, the technique most commonly used at the early diagnosis stage in clinical practice, requires only a small amount of MP-DNA fragments and possesses high sensitivity and specificity of detection. Nevertheless, PCR has high requirements for operators, needs expensive equipment, and requires high standards on sample collection, storage, and processing. Hence, it cannot be broadly applied in major primary hospitals. On the other hand, LAMP has some potential advantages, such as high sensitivity, high specificity, a wide detection range, which is comparable to that of PCR, but is simpler in terms of equipment and instruments used. Moreover, LAMP has high throughput that is achieved fast and without dependence on specialized equipment. Meanwhile, the assay is simple to perform; only one-week training is needed even for technicians with no prior molecular analysis experience. Moreover, the result is convenient to observe, the white turbid or green fluorescence can be directly seen by naked eye. Due to the on the above advantages, *Mycoplasma pneumoniae* can be detected in the early stage of disease. Since the first publication of the official report on this technology by Notomi in 2000, LAMP has been widely used in many life sciences for the detection of pathogens causing diseases, such as malaria, trypanosomiasis, and babesiosis

Nonetheless, the overall clinical accuracy of LAMP for the detection of *Mycoplasma Pneumoniae* has to be further studied. Also, many investigations mentioned that LAMP still has some weaknesses to be overcome. First, LAMP stability is poorer than PCR. Meanwhile, LAMP had a shorter total reaction time.

Due to the limited data reported of the tested samples, the too small sample size, and the lack of comparability, no group evaluation was conducted. The sensitivity and specificity were calculated only based on the number of subjects involved. However, in clinical settings, this part of the data for laboratory tests of different samples is of substantial significance and should be more carefully considered. Hence, this analysis needs to be further improved after the increase of clinical data accumulated in the future.

In summary, LAMP is a method with high sensitivity and specificity when used for the diagnosis of *Mycoplasma Pneumoniae*. LAMP is a rapid, sensitive, and specific detection method that is valuable for the early clinical diagnosis of *Mycoplasma Pneumoniae*. Notably, it can be used to guide the overall clinical treatment and considerably contribute to the prevention of epidemic and complications. Furthermore, it can lead to a reduction in the abusive application of antibiotics; it shortens the duration of treatment, and efficiently improves the initial treatment of patients, decreasing the patient’s pain and economic burden. Along with the advancement of the LAMP technology, LAMP can probably become a primary auxiliary diagnosis choice for *Mycoplasma Pneumoniae* in the near future.

## Conclusions

In summary, we used PCR as the golden standard that have long been applied in the clinical diagnosis of *Mycoplasma Pneumoniae*. Our study provides a valuable reference for another method with high sensitivity and specificity, as well as time efficiency and convenience. However, more clinical data are needed to support our conclusion.

## Abbreviations

AUC: Area under the curve
CI: Confidence intervals
DOR: Diagnostic odds ratio
LAPM: Loop-mediated isothermal amplification
MP: Mycoplasma pneumoniae
NLR: Negative likelihood ratio
OR: Odds ratio
PCR: Polymerase chain reaction
PLR: Positive likelihood ratio
PRISMA: Preferred Reporting Items for Systematic Reviews and Meta-Analyses
QUADAS: Quality assessment of diagnostic accuracy studies
SEN: Sensitivity
SP: Specificity
SROC: Summary receiver operating characteristic
